# New Antiperiodics

**Published:** 1851-01

**Authors:** 


### RECORD OF MEDICAL SCIENCE.

### MATERIA MEDICA AND THERAPEUTICS.

*New Antiperiodics.*—The French medical world has been lately on the *qui vive*, on the subject of antiperiodics, stimulated by a prize of 4,000 francs, offered by the Society of Pharmacy, for the discovery of a substitute for Quinine, and to which the French Minister of war has offered to add an equal sum. M. Delioux, Professor of Materia Medica, at Rochefort, maintains that CHLOROFORM is a powerful succedaneum for cinchona and arsenic. A sufficient number of cases of periodic fevers, which are very common at Rochefort, were treated at the hospital there, with chloroform, and with such a regularity of success, that M. Delioux feels warranted in recommending it as a powerful antiperiodic. The chloroform was given in doses of from nine to thirty grains, according to the severity of the case. The patients took it several times before the access, and continued its use for several days. To make a good mixture, the chloroform is to be first rubbed up with syrup, and then it mixes readily with water. —*Journal de Med. et Chirur. Prat.*, *July*, 1850.

The Physalis Alkekengi, or winter-cherry of France, is also proposed as a remedy for intermittents. The whole plant, twigs, leaves, capsules and berries, are described as possessing the anti-periodic qualities of cinchona—*Gaz. Méd.*, *July*, 1850.

